# Interventions provided in the acute phase for mild traumatic brain injury: a systematic review

**DOI:** 10.1186/2046-4053-2-63

**Published:** 2013-08-07

**Authors:** Jocelyn Gravel, Antonio D’Angelo, Benoit Carrière, Louis Crevier, Miriam H Beauchamp, Jean-Marc Chauny, Maggy Wassef, Nils Chaillet

**Affiliations:** 1 Département de Pédiatrie, CHU Sainte-Justine, Université de Montréal, Montréal, Canada; 2 Département de Chirurgie, CHU Sainte-Justine, Université de Montréal, Montréal, Canada; 3 Département de psychologie, Université de Montréal, Montréal, Canada; 4 Département d’urgence, Hôpital Sacré-Cœur, Université de Montréal, Montréal, Canada; 5 Centre de recherche du CHU Sainte-Justine, Université de Montréal, Montréal, Canada; 6 Section d’Urgence, Département de Pédiatrie, CHU Sainte-Justine, 3175 Chemin Côte Sainte-Catherine, Montréal QC H3T 1C5, Canada

**Keywords:** mTBI, Traumatic brain injury, Head concussion, Systematic review, Treatment

## Abstract

**Background:**

Most patients who sustain mild traumatic brain injury (mTBI) have persistent symptoms at 1 week and 1 month after injury. This systematic review investigated the effectiveness of interventions initiated in acute settings for patients who experience mTBI.

**Methods:**

We performed a systematic review of all randomized clinical trials evaluating any intervention initiated in an acute setting for patients experiencing acute mTBI. All possible outcomes were included. The primary sources of identification were MEDLINE, Embase, PsycINFO, CINAHL, and the Cochrane Central register of Controlled Trials, from 1980 to August 2012. Hand searching of proceedings from five meetings related to mTBI was also performed. Study selection was conducted by two co-authors, and data abstraction was completed by a research assistant specialized in conducting systematic reviews. Study quality was evaluated using Cochrane’s Risk of Bias assessment tool.

**Results:**

From a potential 15,156 studies, 1,268 abstracts were evaluated and 120 articles were read completely. Of these, 15 studies fulfilled the inclusion/exclusion criteria. One study evaluated a pharmacological intervention, two evaluated activity restriction, one evaluated head computed tomography scan versus admission, four evaluated information interventions, and seven evaluated different follow-up interventions. Use of different outcome measures limited the possibilities for analysis. However, a meta-analysis of three studies evaluating various follow-up strategies versus routine follow-up or no follow-up failed to show any effect on three outcomes at 6 to 12 months post-trauma. In addition, a meta-analysis of two studies found no effect of an information intervention on headache at 3 months post-injury.

**Conclusions:**

There is a paucity of well-designed clinical studies for patients who sustain mTBI. The large variability in outcomes measured in studies limits comparison between them.

## Background

Mild traumatic brain injury (mTBI) is defined as the presence of head trauma, a Glasgow Coma Scale (GCS) score of 13 to 15, and at least one of the following four criteria: any period of loss of consciousness; any loss of memory for events immediately before or after the accident; any alteration in mental state at the time of the accident (for example, feeling dazed); or focal neurological deficit(s) that may or may not be transient [1,2].

The WHO estimates that the incidence of mTBI is of 600 cases per 100,000 adults in USA [3-5], with higher incidence for young adults and athletes [3,6-11]. For children, the WHO reports incidences varying from 50 to 100 cases per 100,000 children-years depending on age [3,12-14]. Although most studies suggest that the long-term evolution of mTBI is excellent, with complete resolution of symptoms in 3 months [15-17], 55 to 90% of patients who sustain mTBI experience post-concussion symptoms during the week following the accident [15,18,19]. The nature of these symptoms can be cognitive (memory loss, attention deficit), somatic (headache, fatigue, nausea), or psychological (depression, irritability).

Most patients requiring medical resources secondary to mTBI are initially evaluated at the emergency department (ED). Guidelines pertaining to the management of mTBI generally recommend activity restriction and/or treatment of symptoms [20-23]. Few studies have evaluated potential interventions initiated in the ED for patients who sustain mTBI. A systematic review by the WHO was conducted to evaluate prevalence, outcome, and potential treatment for mTBI [24,25]. Only 16 studies describing potential treatments for mTBI were included. The main limitations of that review were that it included articles published only until the year 2000, and it did not evaluate the effects of interventions on outcomes at 1 week and 1 month. The objective of the current study was to identify all clinical trials of interventions that could be initiated in an acute setting for patients who sustain mTBI.

## Methods

### Design

This was a systematic review of the literature to identify randomized clinical trials evaluating any intervention in the acute phase of trauma versus any comparator or placebo for patients with mTBI.

### Data source and identification of studies

A database search strategy was formulated by one of the authors (MW) who has experience in conducting systematic reviews. A literature search was performed to identify clinical trials and randomized clinical trials including patients with mTBI (GCS score between 13 and 15) seeking acute treatment, and comparing any intervention in the acute phase (first week) of trauma versus any comparator or placebo. Main outcomes measured were somatic post-concussion symptoms (headaches, dizziness, vision, fatigue, irritability, or sleep problems). Other symptoms also considered were cognitive symptoms (memory, attention, concentration, cognition, and language), psychological or emotional symptoms (anxiety, depression, or irritability), autonomy, return to activity (work, school or sport), physical disability, hospital or emergency room readmission, and any side effects of the intervention.

The literature search was conducted via OvidSP in the following electronic databases: Embase, MEDLINE, EBM Reviews, ACP Journal Club, Cochrane Central Register of Controlled Trials, PsychINFO and CinHAL. A Boolean search was constructed using the following MESH terms: ‘head, brain, cerebral, craniocerebral’ combined with any of the following MESH terms: ‘trauma, injury, concussion or post concussion’ and the following MESH term: ‘clinical trial’. Appropriate MESH words were searched and adapted to each database. The search was limited to human subjects and articles published between 1980 and current (August 2012) with no language limitation.

An additional search was conducted in Google Scholar and PubMed, in the references from relevant reviews and clinical trials, and by using authors’ names and searching in similar studies to identify potential additional clinical trials. A hand search of conference proceedings of the International Brain Injury Association, the International Neuropsychological Society, the Society of the American Association of Neurological Surgeon, the Society of Academic Emergency Medicine, and the Canadian Association of Emergency Physicians was also conducted for the years 2010, 2011, and 2012.

Inclusion criteria were: trials including patients, without age restriction, seeking acute treatment after a head trauma, with a GCS score between 13 and 15, and one of the following symptoms:

• Temporary loss of consciousness (less than 30 minutes).

• Amnesia of less than 24 hours.

• Altered state of consciousness.

• Transient focal neurological deficit.

All interventions were eligible. These included, but were not limited to, medications, psychological therapy, patient education (including brochure, pamphlets or meeting with a health professional), activity restriction, hospitalization, or bed rest, and follow-up in a specialized clinic.

Exclusion criteria were: all trials concerning only moderate to severe cases of TBI (GCS <13); clinical trials where time between the trauma and the intervention was more than 1 week; trials concerning only subgroups of patients (for example, patients with insomnia); trials with diagnoses or outcomes relevant only for moderate or severe TBI (for example, death); trials where the extraction of data regarding mTBI was not possible; and studies performed on animals or cadavers.

### Study selection

Three authors (BC, AD, and JG) independently screened in duplicate all titles and abstracts identified by the search. The full manuscripts of all studies selected by at least one reviewer were then evaluated independently and discussed by two authors (BC and JG) to identify the clinical trials to be included. Finally, the full manuscripts of the clinical trials were reviewed independently in accordance with the inclusion and exclusion criteria, and listed to select the final studies included in this review. During these three steps, discrepancies were discussed and resolved by consensus between the authors, otherwise, a third author was consulted to reach an agreement.

### Assessment of risk of bias in included studies

Two authors (JG and MW) assessed the minimum inclusion criteria for randomized clinical trials described in the Cochrane Effective Practice and Organization of Care (EPOC) review group [26]. To be included, a study had to use a proper randomization tool to assign the patient prospectively, have objective measurement of performance, and have relevant and interpretable data presented or obtained. Two authors (JG and MW) independently assessed each study using the Risk of Bias tool for randomized clinical trials described in the method guide for comparative effectiveness [27]. The tool evaluated the following risks of bias: selection (sequence allocation, concealment, and confounding, analysis of patients in the group to which they were randomized), performance (concurrent intervention and fidelity to protocol), attrition, detection (length of follow-up, blinding of assessor, and validity of measurement of intervention, outcomes and confounders), and reporting bias. Each item received a rating of ‘no’ if judged to be at high risk of bias, a rating of ‘yes’ if judged to have a low risk of bias, and ‘unclear’ when the evaluator could not conclude the risk of bias. Any discordance between the authors was discussed and resolved by consensus. Each study was rated as having a low, unclear, or potential risk of bias.

### Data extraction and management

Two authors (JG and MW) independently extracted data from each study using a standardized data-extraction checklist. The database included: demographic data of the study; general information (author, year of publication, year of enrollment in the study, country of the study, setting, study design, randomization tool used, and duration of the follow-up); patient information (number of patients, age, number of patients who dropped out, inclusion and exclusion criteria, and diagnosis of mTBI on admission); GCS score; intervention and comparator description; category and number of patients in each group and outcome description; category and measurement tools; and results of outcome of interest in detail including somatic, cognitive, emotional and ‘return to activities’ data. In cases of missing data or lack of transparency, authors were contacted for clarification. In the absence of a response, the data were considered unattainable. Discordances between reviewers were resolved by consensus. Data were then entered into the Cochrane Review Manager software (RevMan5, 2008) and checked for accuracy.

### Analysis

The large heterogeneity in interventions, outcomes measured, and timing of assessment reported in the studies that met our inclusion criteria limited meta-analytic possibilities. *A priori*, it was decided to conduct a meta-analysis if two or more studies fulfilled the following three criteria: 1) they evaluated a similar intervention; 2) they measured the same outcome; 3) they measured the outcome during the same time frame. Based on the characteristics of the studies identified, two meta-analyses were performed to assess the mid-term effects (1 to 3 months) of information given during the visit to the ED versus routine care, and long-term effects (6 to 12 months) of phone or clinical follow-up versus routine care. Dichotomous data were meta-analyzed, using odds ratios (OR) with 95% CI as measures of effect size, or Peto OR if the number of events in a group was equal to 0. Inter-study variation was incorporated with the assumption of a random effects model for the treatment effect using the DerSimonian and Laird method and the inverse variance method for dichotomous data when heterogeneity between trials was significant or was greater than 50% [26]. In all randomized clinical trials, outcomes were directly compared between the control and the intervention group on an intention-to-treat basis (ITT). The Q and I^2^ tests were used for addressing heterogeneity [26]. If significant heterogeneity was detected, subgroup analyses were carried out by type of study intervention.

### Ethics approval

Because the study did not involve recruitment or assessment of any patients, no research ethics board evaluation was necessary.

## Results

**Figure 1:**
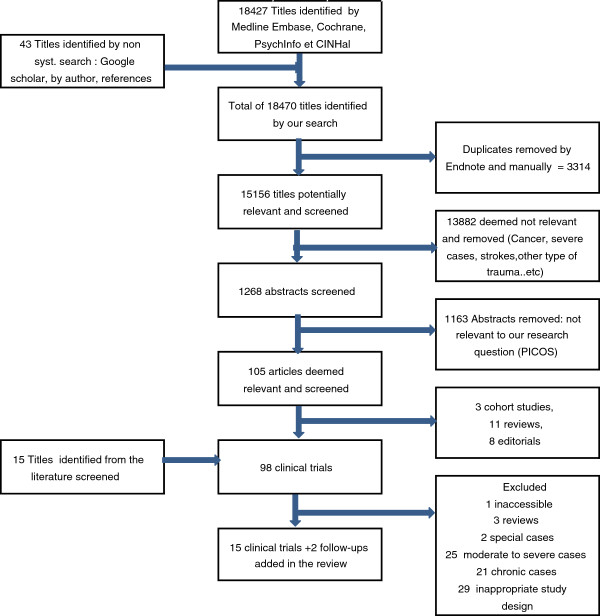
Flow chart of studies.

After removing duplicates, the literature search initially identified 15,156 potential studies (see Figure 1). Most of these were excluded based on their title because they were not related to mTBI. A total of 1,268 abstracts were evaluated, and 1,163 were excluded because they did not meet our inclusion/exclusion criteria. The full manuscripts of 105 articles were evaluated in addition to 15 articles identified through the bibliographies. Of these, 17 articles describing 15 distinct studies fulfilled the inclusion/exclusion criteria. A hand search of the five conference proceedings failed to identify any other potentially pertinent study.

The characteristics of the included studies are described in Table 1. Only one study evaluated a pharmacological intervention [28]. Four studies evaluated standardized information sessions provided in the acute setting with or without an information booklet [29-32]. Seven studies evaluated follow-up interventions compared with no follow-up or routine follow-up [33-39]. Two studies evaluated activity restriction (full bed rest for 6 days [40] and hospital admission for 24 hours [19]). Finally, one study evaluated the long-term outcome of performing a computed tomography (CT) scan of the head versus admission for patients with mTBI [41]. The size of the studies ranged from 17 to 2,602 participants, with a median of 262 participants. Patients of all ages were included. Three studies involved only children, one study involved children and adults, and eleven studies involved only adults or patients older than 15 years. Multiple outcomes were measured at intervals varying between 3 days and 10 years post-trauma.

**Table 1:** Characteristics of the studies

**First author,**	**Inclusion criteria**	**Number of**	**Intervention**	**Outcomes and results**	**Risk of bias**^**a **^**(specific areas of risk of bias)**
**publication year**		**participants**			
	Studies including children only				
Bell [34]	Age <16 years old, mTBI of <48 hours’ duration, n = 366	366	Scheduled phone contact in the first 3 months, standardized instruction handout, and a toll-free phone number CDC booklet (Facts about concussion and brain injury and where to get help), versus usual care	Fewer symptoms and less effect of symptoms on functioning at 6 months for the intervention group according to the post-traumatic symptom composite score (52.6 versus 46.0). No difference in general health composite score	Low
Casey [29]	6 months to 14 years old. minor head trauma but exclusion of patients who loss consciousness	340	Discharge interview during which the nurse explained a take-home booklet of symptoms and phone follow-up carried out the day after discharge, versus usual care	No influence on a list of post-concussion symptoms 1 month after the accident	Potential (unclear for sequence allocation, concealment and blinding. No reporting of confounding and poor outcome measure)
Ponsford [37]	6 to 15 years old, mTBI, GCS 13 to 15	130	Contacted in 48 hours and received neuropsychological assessment in 5 to 7 days plus information booklet, versus no follow-up and no booklet	Less post-concussion symptoms in the intervention group at 3 months	Potential (not randomized, no concealment confounding)
	Studies including adults and children				
Af Geijerstam [41]	> 5 years old, mTBI within the previous 24 hours, GCS of 15	2602	Immediate CT scan of the head versus admission	No statistically significant difference, Glasgow outcome scale not returned to normal at 3 months (21.4% versus 24.2%)	Low
	Studies including mainly adults		Pharmaceutical intervention		
Filipova [28]	18 to 60 years old mTBI	17	Nasal DDAVP (10 μg twice daily) for 5 days versus placebo	Intervention was associated with better results on information-processing test (PASAT) and verbal logical memory after 3 days of treatment. However, no effect seen on four other tests	Low
			Information at discharge		
Hinkle [30]	mTBI or skull fracture, GCS 13 to 15	1092	Standardized information at discharge, versus standardized information plus reassurance plus phone follow-up, versus routine care	Patient return to work and social activities in the information and information plus reassurance group occurred at least 1 week sooner than in the routine treatment group	Unclear (sequence allocation, concealment, blinding and outcome measure)
Mittenberg [32]	Patients admitted for mTBI (adults), GCS 13 to 15	58	A 1 hour meeting with a therapist plus a 10 page manual plus a 10 minute questionnaire, versus routine care	Intervention associated with shorter duration of symptoms (33 versus 51 days) and fewer symptoms at follow-up at 6 months	Unclear (sequence allocation, concealment, blinding and confounding)
Paniak [31]	Adults, mTBI in the previous 3 weeks, exclusion of patients known to have psychiatric disorder	119	Three to four hours of neuropsychological and personality assessment and treatment as needed plus single session with investigator session and a brochure, versus a single session with investigator and a brochure	No effect of intervention on social functioning and SF-36	Low
			Follow-up strategies		
Andersson [33,42]	16 to 60 years old, mTBI	395	Telephone contact at 2 to 8 weeks, follow-up in rehabilitation medicine, and outpatient appointment weekly as needed, versus usual care	No difference in post-concussion symptoms at 1 year or 10 years after mTBI	Unclear (concealment)
Ghaffar [35]	16 to 60 years old, mTBI presenting to the emergency department	191	Follow-up in a multidisciplinary clinic within 1 week and then as needed, and treatment according to specific complaints, versus no follow-up	No effect on the RPCSQ	Unclear (sequence allocation, concealment, confounding, blinding, and fidelity to protocol)
Heskestad [36]	> 15 years old, minimal, mild and moderate TBI	326	Follow-up in neurosurgery clinic within 12 to 17 days after the accident, versus no follow-up	No effect of intervention on post-concussion symptoms	Potential (not randomized. no concealment. 15% completed the study)
Ponsford [39]	> 15 years old, mTBI	262	Contacted in 48 hours and received neuropsychological assessment in 5 to 7 days plus information booklet, versus no follow-up and no booklet	Fewer post-concussion symptoms related to anxiety in the intervention group at 3 months	Potential (not randomized, no concealment confounding)
Wade [38]	16 to 65 years old, head injury of any severity	1156	Approached at 7 to 10 days after injury and offered additional information, advice, support, and intervention as needed, versus no follow-up	No benefit on the RPCSQ at 6 months	Low
			Other interventions		
De Kruijk [40]	> 15 years old, mTBI of 6 hours or more	107	Full bed rest for 6 days followed by gradual mobilization versus gradual mobilization	No effect of bed rest on symptoms secondary to concussion at 2 weeks, 3 months, and 6 months	Unclear (concealment, and fidelity to protocol)
Lowdon [19]	18 to 50 years old, minor head injury with loss of consciousness	114	Admission overnight versus discharge	Admission had no effect on the incidence and had a deleterious effect on the duration of symptoms for 6 weeks	Unclear (sequence allocation, concealment, and fidelity to protocol)

Abbreviations: *CDC* Centers for Disease Control, *CT* computed tomography, *DDAVP* 1-deamino-8-D-arginine vasopressin, *GCS* Glasgow Coma Scale, *mTBI* mild traumatic brain injury, *PASAT* Paced Auditory Serial Addition Test, *RPCSQ* Rivermead Post-Concussion Symptoms Questionnaire, *SF-36* Short Form 36.

^a^Risk of bias criteria according to the Cochrane and EPOC Risk of Bias tool.

**Figure 2:**
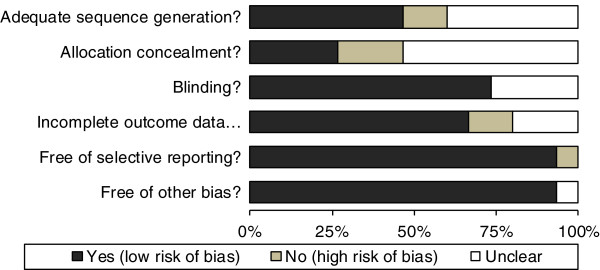
Summary of the risk of bias for the 15 studies.

Of the 15 studies, 5 presented a potential risk of bias. The main reasons for potential biases were related to the randomization sequence generation and the inadequate concealment of allocation of participants (Figure 2). The risk of bias was unclear for six studies. Most of these had unclear information about multiple components of the Risk of Bias tool. The most problematic components were sequence allocation and concealment.

### Interventions

The only pharmacological study reported that, compared with placebo, nasal administration of 10 μg bid of 1-desamino-8-d-arginine-vasopressin (DDAVP) for 5 days was associated with better performance on two memory tests on the third day of treatment for adults who sustained mTBI [28]. The main limitations of the study related to the small sample size (n = 17), the small statistical effect, and the fact that the intervention had no influence on four other cognitive outcomes.

**Figure 3:**
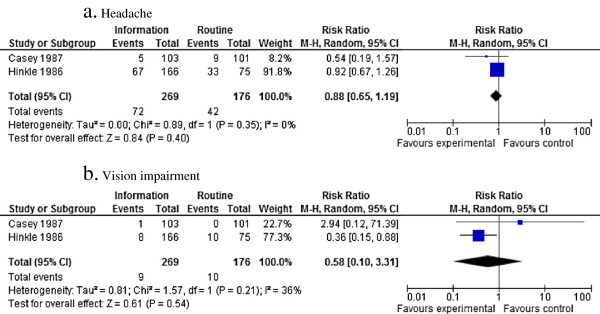
Association between standardized information interventions compared with routine or no information on multiple post-concussion symptoms at 1 to 3 months.

Four studies evaluated a standardized information intervention provided in the ED. Three studies [29-31] reported that the intervention was not more effective than usual care in decreasing post-concussion symptoms, while one study [32] reported that meeting with a specialized therapist and the provision of a 10-page information booklet decreased post-concussion symptoms among adults admitted for mTBI. One study suggested that standardized information and reassurance provided at the ED was associated with faster return to work and social activities [30]. The baseline characteristics of these studies allowed us to conduct a meta-analysis using two studies [29,30] that reported outcomes on individual signs or symptoms secondary to mTBI at 1 to 3 months following a standardized information intervention provided in the acute setting. However, the pooled data failed to show an association between the intervention and the persistence of headache (relative risk (RR) = 0.88; 95% CI 0.65 to 1.19) or vision impairment (RR = 0.58; 95% CI 0.10 to 3.31). The major limitations of this analysis related to the fact that the study population was different for the two articles (adults versus children) and that one of the studies had a potential risk of bias according to our evaluation. A funnel plot is provided in the supplementary material (Figure 3).

**Figure 4:**
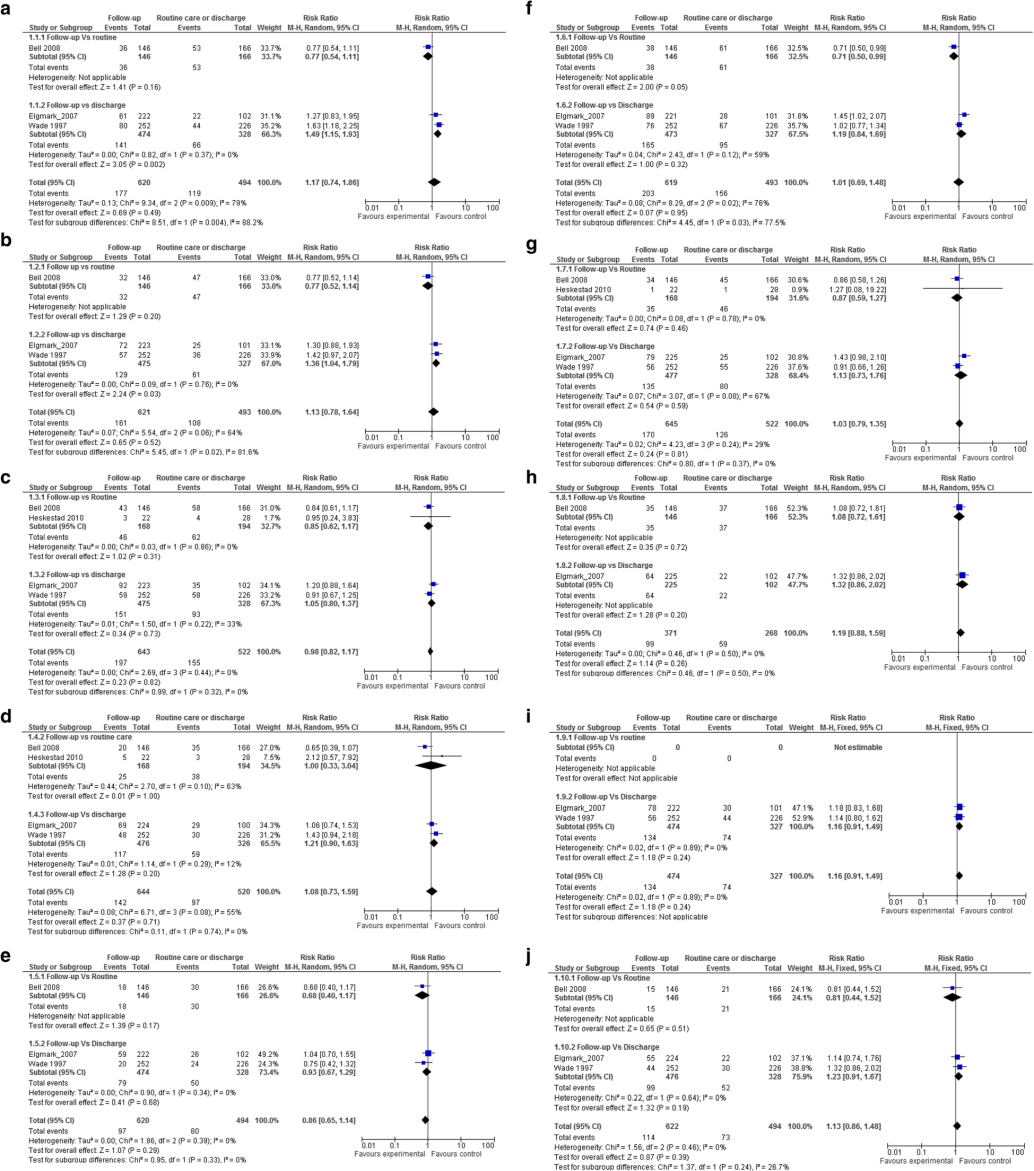
**Association between follow-up interventions compared with routine or no follow-up on multiple post-concussion symptoms at 6–12 months. (a)** Memory, **(b)** poor concentration, **(c)** headache, **(d)** dizziness, **(e)** vision impairment, **(f)** fatigue, **(g)** irritability, **(h)** anxiety, **(i)** depression, and **(j)** sensitivity to noise.

Seven studies evaluated the effect of a follow-up intervention compared with routine or no follow-up [33-39,42]. The interventions were of various types. For example, in one study, the intervention consisted of a phone follow-up, a neuropsychological evaluation at 1 week, and an information booklet [37], while in another study, the intervention was limited to a phone follow-up 2 to 8 weeks after the trauma [33]. Three studies showed a positive effect of the intervention [34,37,39]. These interventions were 1) an information booklet, phone follow-up in 48 hours, and follow-up in a specialized clinic 5 to 7 days after the trauma (evaluated in two studies); and 2) scheduled phone contact in the 3 months after trauma, in addition to an information handout and an information booklet. Importantly, two of these studies were potentially biased according to our evaluation. A meta-analysis was possible using four studies (one with children and three with adults) [33,34,36,38] that reported outcomes on individual signs or symptoms secondary to mTBI at 6 to 12 months after trauma. No association was seen between the intervention and headache (RR 0.98; 95% CI 0.80 to 1.37), poor concentration (1.13; 95% CI 0.78 to 1.64), memory problems (RR 1.17; 95% CI 0.74 to 1.86), dizziness (RR 0.58 95% CI 0.10 to 3.31), vision problems (RR 0.86; 95% CI 0.65 to 1.14), fatigue (1.09; 95% CI 0.69 to 1.48), irritability (1.03; 95% CI 0.79 to 1.35), anxiety (1.19; 95% CI 0.88 to 1.59), depression (1.16; 95% CI 0.91 to 1.49) or sensitivity to noise (1.17; 95% CI 0.74 to 1.86) (Figure 4a-j; see supplementary material). Many of these analyses identified important heterogeneity between studies with I^2^ values greater than 50%. However, secondary analysis of the two studies [33,38] comparing follow-up intervention with no follow-up showed a positive effect for follow-up intervention on the improvement of memory and concentration in adults (Figure 4a,b). Importantly, this sub-analysis showed no heterogeneity between studies.

One large study (n = 2523 participants) failed to show any effect at 6 months for patients with mTBI who had an immediate head CT scan compared with admission [41]. Finally, two studies evaluating different strategies to restrict activity (full bed rest [40] or admission [19]) failed to show a clinical effect at 2 weeks to 6 months.

### Variability in outcomes measured

The reported primary outcomes were mostly related to post-concussion symptoms using various measurement tools (for example, Rivermead Post-Concussion Symptoms Questionnaire (RPCSQ) [43], visual analog scale (VAS), head injury checklist). Some studies reported data for individual symptoms. For example, presence of headache was assessed and reported in nine studies, and memory problems and dizziness were reported in seven studies. Other studies reported only a summary score for symptoms (for example, RPCSQ) or quality-of-life tools (for example, Short-Form Health survey [44], Glasgow Outcome Scale [45]). There was also important variability in the timeframe for outcome measurement. Only four studies reported outcomes measured in the first 2 months after mTBI, and five studies reported only outcomes occurring 6 months after intervention.

## Discussion

This systematic review identified 15 randomized clinical trials evaluating an intervention provided or initiated in an acute setting for patients who sustained mTBI. Our study highlights the paucity of research regarding treatment for mTBI. Moreover, we found that a pharmacological intervention for patients who sustained mTBI has been evaluated in only one randomized clinical trial. This is particularly surprising considering that patients with mTBI represent more than 90% of all TBIs [21]. Our systematic review highlights the important heterogeneity in outcomes measured for patients who sustain mTBI. Although most studies reported many outcomes, no single primary outcome was clearly reported in the majority of studies.

Two systematic reviews of treatments for mTBI have been published previously. The WHO identified sixteen studies of interventions for patients who sustained mTBI [25], of which nine were randomized clinical trials and eight described an intervention that could be initiated at the ED. Three of the identified publications did not fulfill the inclusion criteria for our study: one trial was conducted before 1980 [46], another included only patients with persistent post-concussion symptoms at 3 months [47], and the third included all levels of TBI, from which it was impossible to isolate patients with mTBI [48]. The second systematic review, by Comper *et al*., identified twenty studies (including nine randomized clinical trials) of interventions for mTBI published between 1980 and 2003 [49]. Three of the randomized clinical trials failed our inclusion criteria because they included only patients with post-traumatic stress disorder [50] or headache 1 year after TBI [51], or because it was impossible to isolate only patients who had sustained a mTBI [48]. The conclusion of both reviews was that there was no high-quality intervention study pertaining to mTBI. In addition, their results suggested that a minimal educational strategy that also promotes return to activity as soon as possible might be effective. Our systematic review identified nine new studies not identified by the WHO review and eight not identified in the Compers *et al*. review. The main reason for this is because most of these studies were published after the completion of the previous reviews. Although our systematic review doubles the number of included studies, our conclusions are similar. In addition, it is interesting to note that no new randomized clinical trial on pharmacological treatment for mTBI has been reported for more than 20 years.

More recently, two systematic reviews focused on psychological treatments for mTBI [52,53]. The first review identified eight studies published between 2004 and 2006, in addition to ten studies described in a previous article [52]. Of these eighteen articles, twelve described a randomized clinical trial of which seven described an intervention provided in an acute setting for mTBI. These articles were all identified in our systematic review. This review concluded that there is little evidence to support any active treatment for mTBI but ‘*patient educational approaches may be beneficial if they are initiated in the early period following injury*’ [52]. The second review evaluated all psychological interventions for post-concussion symptoms published until November 2008. The authors identified seventeen randomized clinical trials, of which eight fulfilled our inclusion criteria and were included in our study. The authors concluded that there is some evidence of a positive effect of cognitive behavioral therapy for adults who sustain mTBI, whereas ‘*information, education or reassurance may not be as beneficial as previously thought*’ [53]. These results contrast with two systematic reviews providing evidence to support cognitive rehabilitation of patients who sustain stroke or TBI [54,55]. However, all patients included in the first systematic review had neurological deficits secondary to stroke or TBI [54], whereas the second study included only patients with moderate to severe TBI or patients with mTBI who consulted a rehabilitation clinic for persistent symptoms [55]. These patients do not reflect our study population, and this may explain the difference noted for the effects of cognitive rehabilitation.

The very small number of randomized clinical trials for patients who sustain mTBI is surprising, considering that these patients represent approximately 90% of all patients with TBI. This has an important influence on the research agenda regarding management of patients who sustain mTBI. Even though we identified only one pharmacological study for this study population, emergency physicians frequently prescribe medication for patients who have sustained mTBI [56]. There should be a rigorous evaluation of the mid-term and long-term effects of such medications on decreasing acute symptoms of mTBI. The possible reasons for the lack of publication of pharmacological studies are numerous. Publication bias is not likely to be a factor because no pertinent study was identified by our group in the conference proceedings of five relevant conferences. Rather, there is probably a lack of proof-of-concept research on pharmacological intervention. However, it will be difficult to have an animal model for post-concussion syndrome. Accordingly, clinical researchers should not wait for animal data before suggesting clinical trials for humans who sustain mTBI. Initial studies could evaluate the effects at 1 week of symptom-driven treatment for mTBI. Most guidelines suggest activity restriction after mTBI [57,58], but our systematic review failed to find a positive effect for this type of intervention, and further study is needed in this area.

Our study highlights the wide variety of clinical outcomes reported in the included studies. Furthermore, studies used different measurement tools for the same outcome. For example, post-concussion symptoms were reported individually (for example,: presence or absence of headache) or using a composite score grouping many symptoms (for example,: Rivermead Post-Concussion Symptoms Questionnaire (RPCSQ, VAS for each symptom, head injury questionnaire). Variability was also related to the timing of measurement. While one study evaluated the effect of the intervention at 3 days, others measured it at 6 to 12 months. Variability in outcome may be related to the presence of divergent definitions of persistent post-concussion syndrome. For example, persistent post-concussion syndrome occurs after 3 months according to the DSM-IV-TR [59], but after only 1 month according to the ICD-10 [60]. This variability has been evoked in the past, and authors have suggested standardization [61-63]. Moreover, the NIH, through the National Institute of Neurological Disorders and Stroke (NINDS), developed the Common Data Element (CDE) project (http://www.commondataelements.ninds.nih.gov/#page=Default). The goal of this initiative was to develop data standards for neurology clinical research. The initiative permitted the creation of common definitions and datasets to consistently capture and record outcomes across clinical studies in neurology. In our systematic review, the outcomes most commonly reported were the presence or absence of headache, dizziness, or memory problems.

### Limitations

The most important limitation to our systematic review is related to the large heterogeneity of interventions and outcomes. This limited our ability to group studies together based on intervention. In addition, the wide diversity in outcome measures and timing of their measurement limited our ability to conduct a meta-analysis including most studies. However, this in turn is one of the main findings of this study, further supporting the need to standardize definitions and methods in order to advance research in the field. A qualitative description of the interventions and the studies will help readers come to a better understanding of the subject. As with all systematic reviews, there is an inherent limitation related to publication bias. The small number of studies and their heterogeneity limited our ability to evaluate the extent of publication bias.

## Conclusion

Our systematic review demonstrates a paucity of clinical trials for patients who sustain mTBI. According to the published literature, no intervention initiated acutely has been clearly associated with a positive outcome for patients who sustain mTBI, and there is little evidence suggesting that follow-up interventions may be associated with a better outcome. Considering the high proportion of patients with persistent symptoms after mTBI, it would seem essential to evaluate potential treatments to decrease these symptoms.

Finally, the large spectrum of outcomes evaluated for patients who sustain mTBI limits the possibilities for comparing treatments. Although a composite index score (continuous data) comprising many symptoms secondary to mTBI may be more useful for research purposes, researchers should also collect data on individual symptoms to permit comparison of studies. In addition, researchers should measure outcomes at 1 week, 1 month and 3 months after trauma. One potential research idea may be to ask patients having sustained a mTBI what outcomes would be meaningful for them.

## Abbreviations

CDC: Centers for Disease Control; CT: computed tomography; DDAVP: 1-deamino-8-D-arginine vasopressin; DSM-IV-TR: *Diagnostic and Statistical Manual of Mental Disorders*, fourth edition, tenth revision; ED: Emergency department; GCS: Glasgow Coma Scale; ICD-10: International Statistical Classification of Disease and Related Health Problems, 10th edition; ITT: Intention-to-treat; mTBI: Mild traumatic brain injury; PASAT: Paced Auditory Serial Addition Test; RPCSQ: Rivermead Post-Concussion Symptoms Questionnaire; SF-36: Short Form 36; VAS: visual analog scale; WHO: World Health Organization.

## Competing interests

The authors declare that they have no competing interests.

## Authors’ contributions

JG conceived the study and obtained funding. All collaborators contributed to the design. ADA, BC, JG, and MW participated in the study selection and evaluated the articles. MB, JMC, LC, and JG performed the manual search of the conference proceedings. NC and MW performed the primary analysis of the data. JG drafted the manuscript, and all authors contributed substantially to its revision. JG takes responsibility for the paper as a whole. Each author listed on the manuscript has seen and approved the submission of this version of the manuscript and takes full responsibility for the manuscript.
